# Spatiotemporal distribution of juvenile chum salmon in Otsuchi Bay, Iwate, Japan, inferred from environmental DNA

**DOI:** 10.1371/journal.pone.0222052

**Published:** 2019-09-04

**Authors:** Yuki Minegishi, Marty Kwok-Shing Wong, Takashi Kanbe, Hitoshi Araki, Tomomi Kashiwabara, Minoru Ijichi, Kazuhiro Kogure, Susumu Hyodo

**Affiliations:** 1 International Coastal Research Center, Atmosphere and Ocean Research Institute, The University of Tokyo, Otsuchi, Iwate, Japan; 2 Atmosphere and Ocean Research Institute, The University of Tokyo, Kashiwa, Chiba, Japan; 3 Research Faculty of Agriculture, Hokkaido University, Sapporo, Hokkaido, Japan; University of Hyogo, JAPAN

## Abstract

To understand the ecology of juvenile chum salmon during early marine life after their downstream migration, we developed a quantitative PCR-based environmental DNA (eDNA) method specific for chum salmon and investigated the spatiotemporal distribution of eDNA in Otsuchi Bay, Iwate, Japan. Indoor aquarium experiments demonstrated the following characteristics of chum salmon eDNA: (1) the eDNA shedding and degradation were time- and water temperature-dependent and the bacterial abundance could contribute to the eDNA decay, (2) fecal discharge may not be the main source of eDNA, and (3) a strong positive Pearson correlation was found between the number of juveniles and the eDNA amounts. As we discovered strong PCR inhibition from the seawater samples of the bay, we optimized the eDNA assay protocol for natural seawater samples by adding a further purification step and modification of PCR mixture. The intensive eDNA analysis in the spring of 2017 and 2018 indicated that juvenile chum salmon initially inhabited in shallow waters in the shorefront area and then spread over the bay from January to June. The eDNA data also pointed out that outmigration of juvenile chum salmon to open ocean temporarily suspended in April, possibly being associated with the dynamics of the Oyashio Current as suggested by a previous observation. The eDNA method thus enables us large-scale and comprehensive surveys without affecting populations to understand the spatiotemporal dynamics of juvenile chum salmon.

## Introduction

Anadromous chum salmon *Oncorhynchus keta* is one of the most economically important fish in northern Japan. Under the hatchery-based stock enhancement program since 1880s, about 1.8 billion juveniles have been annually raised in Japan from homing adults, and released into natural waters in the following spring [1]. This resulted in the remarkable increase of the returning adults, presumably in combination with favorable oceanic conditions and technical improvement in artificial reproduction and juvenile rearing [2]. However, after the peak of more than 80 million returning adults in 1990s, the catch has been decreasing to 30–40 million despite approximately constant annual release of juveniles [1].

The fluctuation of chum salmon populations was shown to be affected by large-scale climate and oceanographic changes [3–7]. For example, Kaeriyama et al. [7] explained the dynamics of chum salmon biomass by the strength of Aleutian Low and the decadal oscillation of the Pacific Ocean that causes the changes in carrying capacity of the environment. On the other hand, coastal environmental conditions such as water temperature have also been suggested to influence the successful growth and outmigration of juveniles. These factors are thought in turn to affect the returning rate of adult chum salmon [8,9]. Saito et al. [10] examined the relationships among the adult returning rate, juvenile coastal growth, juvenile size at release and sea surface temperature. They showed direct associations among them, probably through survival rate of juveniles [10]. These studies strongly indicate the importance of the early marine life of juvenile chum salmon for sustainable and highly-efficient adult return.

In Japan, the Hokkaido and Sanriku regions (the Pacific Ocean side of the northern Japan’s mainland) are the two major homing areas of chum salmon. Juveniles produced in hatcheries are released to natural rivers from February to May when they are sized at 30–50 mm of fork length (FL). Then, they migrate downstream to the sea after relatively short periods in riverine life from one day to few weeks [11,12]. During their downstream migration, juveniles stay at brackish areas in the estuary for some time for osmoregulatory adjustment [13], and then move to coastal waters before outmigration to the Okhotsk Sea. The coastline of the Sanriku region, referred as the Sanriku-ria Coast, is characterized by a large number of small closed bays into which one or several short rivers flow. This is contrasting with the smooth coastline of Hokkaido and other North Pacific countries where long rivers flow directly into the open ocean. In Sanriku, therefore, downstream-migrated juveniles of 40–50 mm FL spend a couple of months in bays, instead of rivers, until their body size reach 80–120 mm FL and/or when the sea surface temperature rises to around 13°C [12,14]. Considering the relationship between the adult return and the coastal phase of juveniles mentioned above, it is important to understand the ecology of juvenile chum salmon in the Sanriku bays.

Traditional methods for ecological studies of juvenile chum salmon such as net sampling and visual inspection often require enormous amounts of efforts. Frequent massive net sampling in wide areas of the bay also has a risk of affecting juvenile populations. In contrast, environmental DNA (eDNA) can be a powerful tool to deduce the spatiotemporal distribution of organisms without collection of organisms themselves. The eDNA technique clearly showed the distribution [15–17], biomass [18–20], life history events such as spawning [21] and species composition in aquatic systems [22–24]. Moreover, the use of eDNA is advantageous also for its sensitivity. An early work using the eDNA technique powerfully detected an alien invasive species of the American bullfrog in eleven ponds in France in which this species was not observed before [15].

Field surveys for juvenile chum salmon in nature across seasons are labor-intensive and demanding, which is one of the reasons that the ecology of juvenile chum salmon before outmigration to the open ocean is poorly understood in the Sanriku area. The aim of the present study was, therefore, to reveal the spatiotemporal distribution of juvenile chum salmon in the bay across the seasons to obtain most basic information on their early marine life. To this end, we utilized the eDNA technique and established a PCR-based method for specific detection of chum salmon eDNA. Then, we performed the intensive water sampling across the seasons for two years and analyzed the spatiotemporal distribution of chum salmon juveniles by the eDNA technique.

## Materials and methods

### Ethics statement

All animal experimental procedures in this study were carried out in accordance with the guidelines for the care and use of animals approved by the animal care and use committees of The University of Tokyo. The fish used for the experiments were not sacrificed during and after the experiment and were put back to the rearing aquarium after experiments. No specific permissions were required for water sampling in the field, since the survey area in the present study is not protected or regulated, and does not involve any endangered or protected species.

### Animals for aquarium experiments

Fertilized eggs of chum salmon were provided by Tsugaruishi salmon hatchery, Iwate, Japan. Eggs were artificially fertilized using adult salmons returned to the Tsugaruishi River during the 2015–2016 and 2016–2017 seasons. Embryos of eyed egg stage were transferred to Atmosphere and Ocean Research Institute, The University of Tokyo, Kashiwa, in late February and kept in natural seawater at 12°C in the dark until yolk-sac absorption. After emergence from the bottom, juveniles were kept in round tanks (250 L) with running recirculating natural seawater at 12°C under a 14h:10h light dark cycle, and fed at 0.5 g of commercial fishmeal per g fish twice a day until experiments following the method established by Leitritz and Lewis [25].

### Aquarium experiment 1: Effect of feeding on eDNA amounts

Rectangular aquaria (34 cm × 20 cm × 21 cm) containing 8 L of artificial seawater, which was made with tap water in a bleached container, were placed in a larger pool with a controlled temperature at 14°C in triplicate for each experimental group. For three days prior to the experiment, fish were acclimated to the experimental water temperature, and one group (“fed” group) was fed about 0.5 g per g fish with commercial fishmeal for two times a day whereas another (“fasted” group) was fasted. A water sample of 150 mL was taken before fish introduction (t = 0). Five juvenile chum salmon were transferred to aquaria and 150 mL of water was sampled in duplicate at 2, 6, 24 and 48 hours. Afterwards, fish were gently removed from each aquarium and water was continuously sampled 1, 2, 3 and 5 days after fish removal. During this experiment, water was continuously aerated but not filtered. Fish were exposed to a 12h:12h light dark cycle during the experiment. Aquaria and sampling containers were washed with neutral detergent, rinsed with tap water, bleached by submerging in solution of 10% bleach for at least 30 minutes, washed with tap water, rinsed with Milli-Q water, and air-dried before use.

### Aquarium experiment 2: Time- and temperature-dependent changes in eDNA amounts

Rectangular aquaria (34 cm × 20 cm × 21 cm) were filled with 8 L of artificial seawater and placed to temperature-controlled larger containers that were set at 8, 14 and 20°C. These water temperatures, 8, 14 and 20°C, were close to the lower limit, optimal and near the lethal limit for juvenile chum salmon, respectively [26]. After sampling of 150 mL of water from each aquarium before fish introduction (t = 0), five juvenile chum salmon (ca. 80 mm FL, ca. 4.0 g of body weight), which were fasted for three days prior to the experiment and acclimated to each experimental temperature, were randomly assigned to aquaria of the same water temperature. At 0.5, 1, 2, 6, 12, 24 and 48 hours after fish introduction, 150 mL of water was sampled in duplicate. After the sampling at 48 hours, fish were gently removed from each aquarium and then water was sampled at every 24 hours up to 5 days. During this experiment, water was continuously aerated but not filtered. Fish were unfed and exposed to a 12h:12h light dark cycle. The experiment was performed in triplicate aquaria. Aquaria and sampling equipment were decontaminated as described above prior to the experiment.

### Aquarium experiment 3: Fish density

Two round aquaria (75 cm in diameter) containing 50 L of artificial seawater were settled in a temperature-controlled room at 14°C, and 3, 10, 30, 60 and 120 fish that were kept at the experimental temperature in an acclimation tank (250 L) for three days prior to the experiment were introduced to the aquaria. After two and six hours after fish introduction, 1 L of water was sampled from each aquarium. During this experiment, water was continuously aerated but not filtered. Fish were unfed and exposed to a 12h:12h light dark cycle during the experiment. Sampling equipment was decontaminated as described above prior to the experiment.

### Species specific primers and probe design

Chum salmon-specific primers and probe for a quantitative real-time PCR (qPCR) were designed in the mitochondrial DNA control region by using the sequence data from the NCBI nucleotide database as follows; a forward primer, OnKeta-spDL-F3, 5'-CCCGCACATTTGTAAATGC-3'; a reverse primer, OnKeta-spDL-R3, 5'-TGATGTATGAGGGGTTAAAATAAG-3'; the probe, OkDL-Probe1, 5'-CCCATATATAATACTGCACGTGAGTAGTAC-3'. Sequence variations in the target region of 163 sites including the priming regions were examined with the DNA sequence of other salmonid species *Oncorhynchus masou*, *O*. *mykiss* and *Salmo trutta* that co-occur with chum salmon in the northern Japan’s mainland. Chum salmon-specific qPCR amplification was tested by using the tissue-oriented DNA samples from chum salmon and these three salmonid species.

### Water filtration, DNA extraction and qPCR assay

All filtration equipment such as funnels and tweezers was treated with a decontamination procedure before sample filtration as described above. Prior to filtration of sample water, 500 mL of Milli-Q water was filtered through a 47-mm diameter glass microfiber filter (GF/F grade; Whatman GE Healthcare Life Science, Buckinghamshire, UK) as negative control of each water sample. Sampled water was then filtered using the same funnel. The glass microfiber filter disk was fixed by 70% ethanol for one minute and immediately stored at -20°C until DNA extraction.

DNA extraction was performed using DNeasy Blood and Tissue kit (Qiagen, Hilden, Germany) as described in Yamamoto et al. [20]. Entire DNA was eluted in 110 μL of elution buffer and stored at -20°C until qPCR.

For eDNA quantification of juvenile chum salmon, we employed either FAM-TAMRA TaqMan probe or FAM-ZEN-IBFQ probe with the identical sequence and performed qPCR with an ABI 7900HT real-time PCR system (Applied Biosystems, Foster City, CA, USA) in a 10-μL of a total volume containing 1×TaqMan Universal Master Mix II (Applied Biosystems, Foster City, CA, USA), 900 nM of each of forward and reverse primers, 250 nM of fluorescent probe and 2.5 μL of template eDNA. The thermal-cycling profile was 50 cycles of denaturation at 95°C for 15 sec, annealing and extension at 60°C for 1 min, preceded by an activation step at 50°C for 2 min and a denaturation step at 95°C for 10 min. The DNA fragment consisting of the target amplicon and priming sites was amplified from genomic DNA and inserted onto a plasmid, which was used as the standard of qPCR (10^1^–10^7^ copies per reaction). All samples were assayed in triplicate.

Bacterial 16S rRNA gene copy numbers in the sampled water were also quantified by qPCR [27]. Briefly described, PCRs were performed on a LightCycler 480 Real-Time PCR System (Roche Applied Science, Mannheim, Germany) with a 20-μL of reaction volume containing 1×Premix Ex Taq (Probe qPCR) (TaKaRa, Shiga, Japan), 100 nM of each primer and 100 nM of dual labeled probe (FAM/BHQ1), and 1 μL of template DNA. A temperature profile was denaturation at 95°C for 3 min, 45 cycles of denaturation at 95°C for 10 sec, annealing and extension at 60°C for 40 sec, followed by cool at 50°C for 30 sec. The DNA fragment containing the target amplicon and priming sites was amplified from environmental sample and cloned into plasmid. This plasmid was linearized by digesting with restriction enzyme and used as standard of qPCR by serial dilution (6.03×10^0^–6.03×10^6^ copies per reaction). All samples were assayed in triplicate.

All statistical tests were carried out using GraphPad Prism version 6 for Windows (GraphPad Software, La Jolla, CA, USA). For the aquarium experiments of the feeding effect and temporal changes in salmon eDNA and bacterial gene amounts, two- and one-way ANOVA followed by the Tukey's multiple comparison tests were performed, respectively. A Pearson’s correlation coefficient test was performed on the relationship between fish density and salmon eDNA amounts. *P*<0.05 was considered as statistically significant.

### Field application

To confirm that our eDNA method established through the indoor aquarium experiments enables to detect the chum salmon eDNA in natural water, 1-L surface water samples were collected in the 2016 spring (April 26 and 27, May 10 and 11) at Murohama of Otsuchi Bay, Iwate, Japan, where the presence of juvenile chum salmon was shown by either beach seine or surf-zone seine net towing.

In the next spring of 2017, 1 L of surface seawater was sampled in duplicate basically every week from early April to the middle of June at five shorefront sites (Hakozaki, Murohama, Murohama fishing port, the river mouth of the Otsuchi River and Hyotan Island) to investigate the spatiotemporal distribution of juvenile chum salmon in Otsuchi Bay. One liter of seawater was also sampled in duplicate at five fixed sampling sites in the bay (Stn. e1 –e4 and Ex. 1) from early May to the middle of June. With regard to these five bay sites, one-, five- and twenty-meter depths of water were sampled using a 12-L Niskin sampler.

In 2018, more intensive water sampling was conducted to examine the temporal changes in the horizontal distribution of juvenile chum salmon in Otsuchi Bay from their sea entry to outgoing to open ocean. To this end, 1-L water sampling in duplicate was carried out basically once a week at the above-mentioned five shorefront sites, five bay sites, and additional three bay sites (Stn. 2, 9 and 11) from the end of January to late June for nearly half a year. Water column temperature and salinity were measured using a CTD sensor (ASTD102, JFE Advantech, Hyogo, Japan) at each sampling site.

All water sampling equipment was decontaminated prior to water sampling as described earlier. Water samples containing benzalkonium chloride at a final concentration of 0.01% [28] were transferred to International Coastal Research Center in Otsuchi in a cooler box with blue ice, and then immediately filtered as stated above. All filter disc samples were stored at -20°C until DNA extraction.

Since strong PCR inhibition was observed in some field samples collected in the spring of 2016 (see the Results section for detail), we tested (1) the purification of the extracted DNA using either Zymo OneStep PCR Inhibitor Removal kit (Zymo Research, Irvine, CA, USA) or DNeasy PowerClean Pro Cleanup Kit (Qiagen, Hilden, Germany) following the manufactures’ instruction, (2) the addition of bovine serum albumin (BSA) to reaction mixture of qPCR assays and (3) the use of qPCR master mix specialized for environmental samples (TaqMan Environmental Master Mix 2.0; Applied Biosystems, Foster City, CA, USA), to achieve an inhibition-free assay condition. By combining these tests, the assay protocol was optimized for the natural seawater samples from Otsuchi Bay, which was then applied for the field samples collected in 2017 and 2018.

To compare the vertical distribution of chum salmon eDNA at different layers, two-way ANOVA was performed. For the horizontal distribution of chum salmon eDNA, only the data from the 2018 spring was used due to the limitation of sample numbers in 2017. For the statistical analyses, a total of 13 sampling sites were divided into three categories for a Chi-square test; Hakozaki, Murohama, Murohama fishing port, the river mouth of the Otsuchi River and Hyotan Island were grouped as ‘shorefront’, Stn. e3, e4, 9, 11 and 2 as ‘inner bay’ and Stn. e1, Ex.1 and e2 as ‘bay mouth’. The seasonal changes in chum salmon eDNA distribution was examined using one-way ANOVA followed by the Tukey's multiple comparison test among the three areas. Unmatched data lacking samples from the bay mouth group were not used in statistical analysis. *P*<0.05 was considered as statistically significant. All statistical tests were carried out using GraphPad Prism version 6 for Windows (GraphPad Software, La Jolla, CA, USA).

## Results

### Species specificity of primers and probe for qPCR

Multiple mismatches were found in the target region of the sequences of the four salmonid species (S1 Fig). The assay designed for chum salmon successfully amplified the DNA of chum salmon, but not the other three salmonid species that are found in the northern Japan.

### Aquarium experiment 1: Effect of feeding on the eDNA amounts

**Fig 1:**
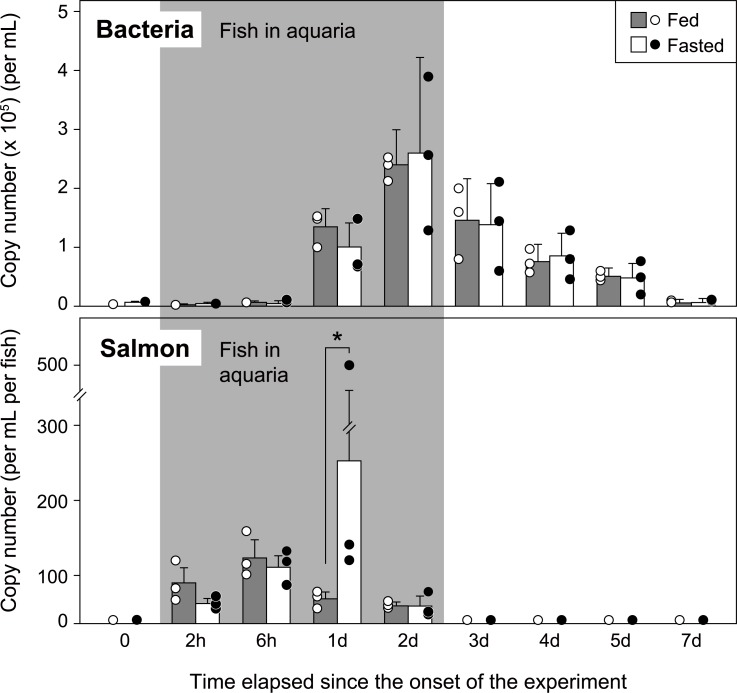
**Temporal changes in chum salmon eDNA (lower panel; per mL water sample per fish) and bacterial gene amounts (upper panel; per mL water sample) in aquaria for the fed (shaded bars and white circles) and fasted groups (white bars and black circles).** Bars and circles present the average and each value of the triplicate aquaria, respectively. Juvenile chum salmon was in aquaria during the shaded period. h and d indicate hours and days, respectively. An asterisk indicates a statistical significance (*P*<0.05).

In the fed group, the largest chum salmon eDNA amount was observed at six hours after fish introduction (Fig 1). The eDNA amount of the fasted group maximized at one day after fish introduction with an extremely high value that was found in one of the triplicated experimental aquaria. Except for that, no statistically significant differences were observed in the copy numbers between the two groups (two-way ANOVA followed by Tukey's multiple comparison, *P*<0.05; Fig 1). Afterwards, the amount of salmon eDNA decreased and was almost undetectable in both groups at three days since the onset of the experiment (i.e., one day after fish removal). The eDNA degradation begun before fish removal in both groups.

Changes in the bacterial gene amounts were also similar between the fed and fasted groups (two-way ANOVA followed by the Tukey's multiple comparison, *P*>0.05; Fig 1). In both groups, the bacterial gene amounts maximized at two days after fish introduction and then decreased (Fig 1).

### Aquarium experiment 2: Time- and temperature-dependent changes in eDNA amounts

**Fig 2:**
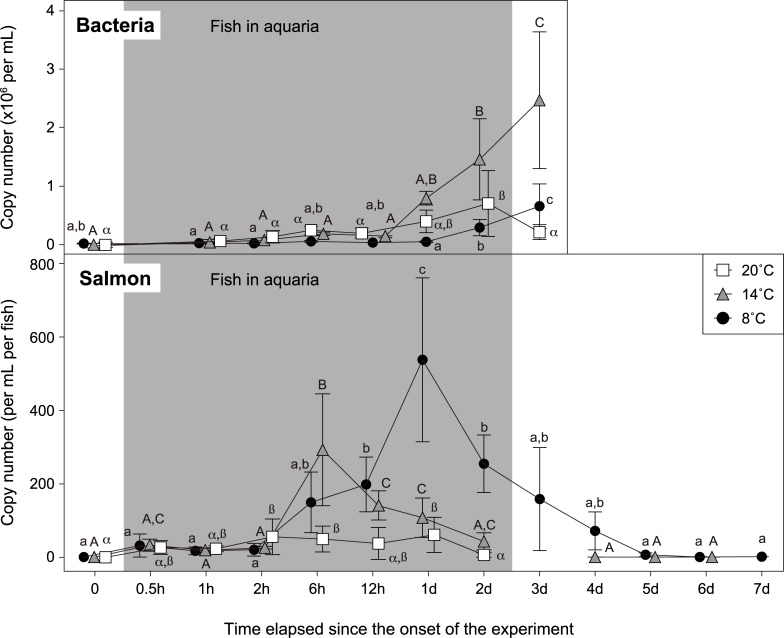
Temporal changes in chum salmon eDNA (lower panel; per mL water sample per fish) and bacterial gene amounts (upper panel; per mL water sample) in aquaria at various water temperatures. Samples at t = 0 were obtained before fish introduction into aquaria. Juvenile chum salmon was in aquaria during the shaded period. Statistical significances were presented with different characters of small, capital and Greek alphabets for 8, 14 and 20°C, respectively (*P*<0.05). h and d indicate hours and days, respectively.

During the experiment, no mortality was observed at any temperature conditions. The chum salmon eDNA amounts were increased after fish introduction and then started to decrease; the patterns of salmon eDNA increase and decrease were diverse among the rearing temperatures (Fig 2). At 8°C, the eDNA amount in aquaria was gradually increased and reached a peak level one day after fish introduction and then decreased even before fish removal. At 14°C, the peak eDNA amount was detected at six hours after fish introduction and begun to decrease thereafter. At 20°C, the amount of eDNA in water peaked at two hours after fish introduction, much earlier than the other two temperatures, but the maximum level was considerably lower than those at 8°C and 14°C. The eDNA levels at 20°C fluctuated and returned to nearly background level two days after fish introduction (Fig 2). One-way ANOVA followed by the Tukey's multiple comparison on time effect showed statistically significant changes between time points at each temperature (*P*<0.05.)

After fish were removed, the chum salmon eDNA amount was further decreased at 8°C and came back to an almost background level three days after (i.e., five days since the onset of the experiment in Fig 2). At 14°C and 20°C, salmon eDNA was undetectable after one day following fish removal.

The bacterial gene amount in the water at 8°C stayed at a low level up to one day after fish introduction, and gradually increased afterwards. At 14°C, the lag phase of increase in the bacterial gene amount was up to 12 hours after fish introduction followed by a rapid increase. At 20°C, the change in the bacterial gene amount was at intermediate levels between those of 8°C and 14°C, in which the eDNA amount was increased gradually after fish introduction, and decreased after fish removal (Fig 2). As well as the results of salmon eDNA, one-way ANOVA followed by the Tukey's multiple comparison on time effect showed statistically significant changes between time points at each temperature (*P*<0.05).

### Aquarium experiment 3: Fish density

**Fig 3:**
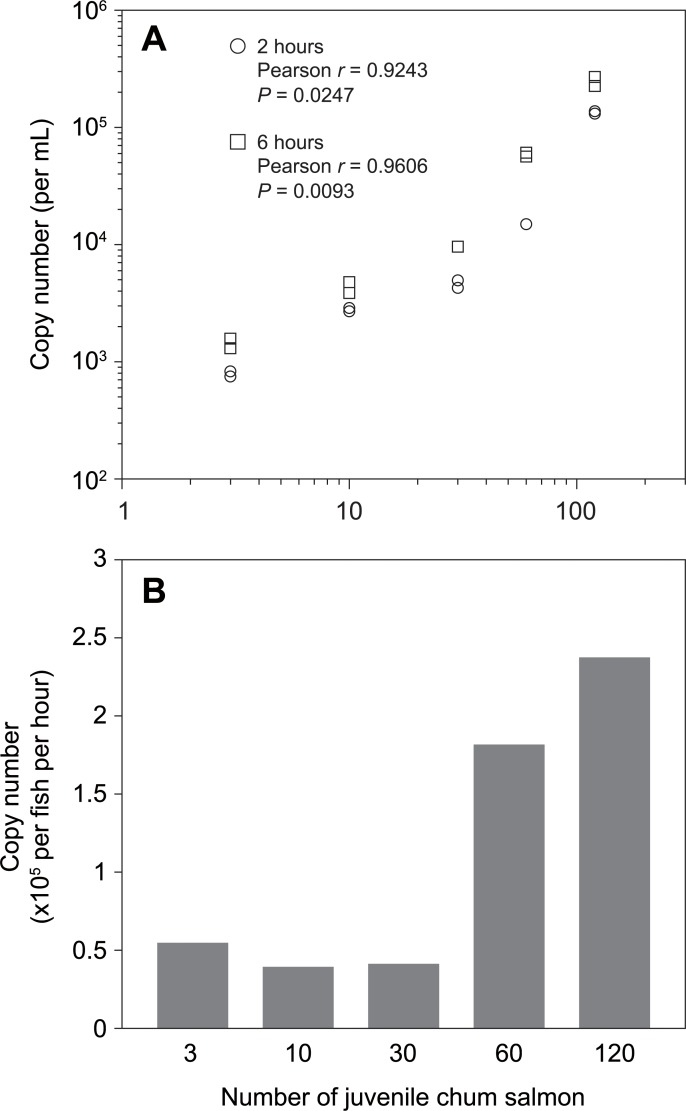
Relationships between the number of juvenile chum salmon and the eDNA amount. A, copy number per mL at two and six hours; B, the eDNA release rates per fish per hour.

A positive correlation between the number of juvenile chum salmon and eDNA copy number was observed in both two and six hours after fish introduction (Pearson *r* = 0.9243, *P* = 0.0247 at two hours; Pearson *r* = 0.9606, *P* = 0.0093 at six hours; Fig 3A). Since large amounts of salmon eDNA were released during the first two hours probably due to the fish transfer into the experimental aquaria from the holding tank, the amounts of eDNA at six hours were subtracted by those at two hours to calculate the eDNA release between two and six hours. The released eDNA amounts from a single juvenile per unit time was calculated using the following formula;

C=(B‐A)/4/n

where C is the eDNA copy number per fish per hour, B and A are the eDNA copy number released at six and two hours, respectively, and n is the number of juveniles in an aquarium. The eDNA copy number per fish per hour was nearly stable among 3, 10 and 30 fish in an aquarium (5.45×10^4^, 3.89×10^4^, 4.19×10^4^ copies per fish per hour, respectively), while that for 60 and 120 juveniles were considerably higher (1.82×10^5^ and 2.38×10^5^ copies per fish per hour, respectively; Fig 3B).

### Field application

When we initially measured the natural seawater samples obtained from Murohama of Otsuchi Bay in the spring of 2016, we failed to detect salmon eDNA, although the existence of many juvenile chum salmon near the sampling site was confirmed by net towing. Using a serial dilution of these water samples into which known amounts of salmon DNA was spiked, we found that the water samples from Otsuchi Bay were highly contaminated with PCR inhibiting substance(s) that prevent PCR amplification. The DNeasy Blood and Tissue kit (Qiagen, Hilden, Germany) alone were unable to remove inhibitor(s), and the inhibition was too strong to resolve this problem by diluting water samples 8,912 times to eliminate PCR inhibition (Table 1; S2 Fig). To remove the PCR inhibitor(s) from the samples, we tested two commercial DNA purification kits and found that the use of DNeasy PowerClean Pro Cleanup kit (Qiagen, Hilden, Germany) significantly reduced the inhibition, but strong inhibition still remained; a 512-fold dilution of water samples was necessary for inhibition-free PCR amplification (Table 1). For samples without purification, the addition of bovine serum albumin (BSA) into the PCR mixture of Universal Master Mix II (ThermoFischer Scientific, Waltham, MA, USA) significantly improved PCR efficiency from an 8,912-fold to a 32-fold dilution to achieve inhibition-free amplification. The use of Environmental Master Mix 2.0 (ThermoFischer Scientific, Waltham, MA, USA) further improved the minimal dilution ratio to 8-fold (Table 1). The use of Environmental Master Mix 2.0 achieved higher sensitivity and higher amplification intensity compared to the use of Universal Master Mix II with BSA. Based on the above results, we finalized the eDNA protocol with sequential DNA extraction by DNeasy Blood and Tissue kit, purification with DNeasy PowerClean Pro Cleanup kit, and real-time PCR amplification using Environmental Master Mix 2.0 for all field samples obtained from Otsuchi Bay, and succeeded to detect the chum salmon eDNA from the samples of the 2016 spring.

**Table 1:** Tested combination of purification methods and different qPCR master mixes for optimization of qPCR assay protocol of chum salmon eDNA collected from Otsuchi Bay.

		PCR master mix		
		ABI TaqMan Universal Master Mix II	ABI TaqMan Universal Master Mix II + BSA (1 mg/mL)	ABI TaqMan Environmental Master Mix 2.0
Purification	None	8,912	32	8
	Zymo Research OneStep^™^ PCR Inhibitor Removal Kit	8,912	ND	ND
	DNeasy PowerClean Cleanup Kit	512	0	0

Numbers indicate the fold dilution required to obtain the inhibition-free qPCR assays. ND denotes no data.

### Spatiotemporal distribution of juvenile chum salmon eDNA

**Fig 4:**
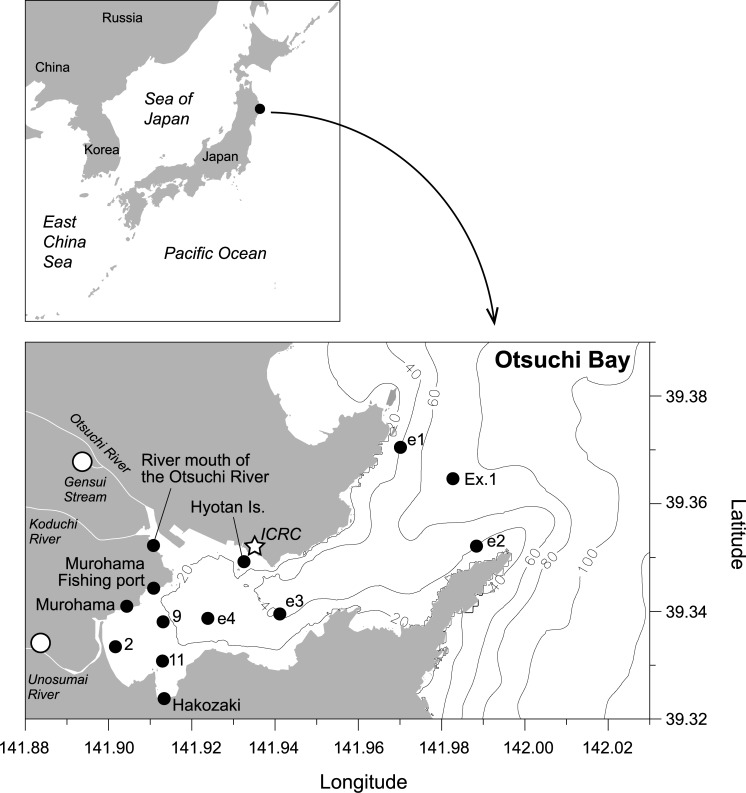
Map showing Otsuchi Bay and the water sampling sites (black circles). A white star indicates International Coastal Research Center where water filtration was conducted. Three rivers (the Otsuchi, Koduchi and Unosumai Rivers) flow into the bay. White circles indicate the salmon hatcheries that released juveniles into the Gensui Stream (consequently, the Otsuchi River) and the Unosumai River, respectively, from February to May.

**Fig 5:**
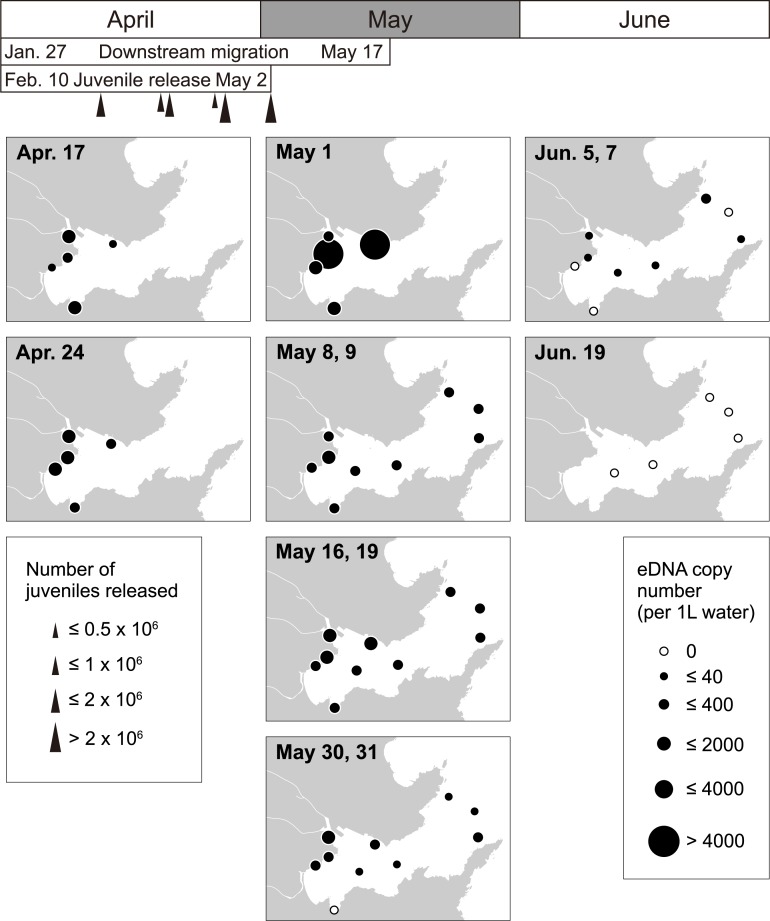
Changes in the horizontal distribution of juvenile chum salmon eDNA in Otsuchi Bay from mid-April to mid-June in 2017. Months are indicated at the top of the panels. The periods of downstream migration in rivers and juvenile release from hatcheries were presented below the months. Triangles below the period showing the juvenile release present the timing and number of juveniles released from two hatcheries. Sampling dates are shown at the top left of each panel and time goes from top to down in each month. In the case that two sampling dates are indicated, water collection was performed in the same week and the results of the samples collected from the shorefront and bay sites were demonstrated in a single panel. At the central bay and bay mouth sites (Stn. e1 –e4 and Ex.1), the data from one-meter depth was shown. White circles present no chum salmon eDNA was detected. The sizes of black circles correspond to the copy numbers. Refer Fig 4 for the detailed locations and names of the sampling sites.

In Otsuchi Bay, three rivers, the Otsuchi, Koduchi and Unosumai Rivers, flow into the bay, and chum salmon juveniles produced in hatcheries were released into the Otsuchi and Unosumai Rivers (Fig 4). In the spring of 2017, chum salmon eDNA was detected at almost all sampling sites from the middle of April to early June (copy number = 5.2–11621.0 per L of sample water; Fig 5; S1 Data). At the shorefront sites (Hakozaki, Murohama, Murohama fishing port, river mouth of the Otsuchi River and Hyotan Is.), relatively higher copy numbers of chum salmon eDNA were found from late April to the middle of May. In this year, juvenile release from the Otsuchi and Unosumai hatcheries was reported to be continued until May 2 (S1 Table), and downstream migration of juveniles was confirmed until May 17 in the Otsuchi River (Kawakami et al. in prep.). From early May, water sampling was conducted also in the central bay and bay mouth sites (Stn. e1 –e4 and Ex.1), and chum salmon eDNA was detected at one-meter depth until early June except for Ex.1 site in early June (June 7). In late May and early June, detectable levels of chum salmon eDNA were not observed at some shorefront sites such as Hakozaki and Murohama. In the middle of June, no eDNA was detected at all sampling sites (Fig 5).

**Fig 6:**
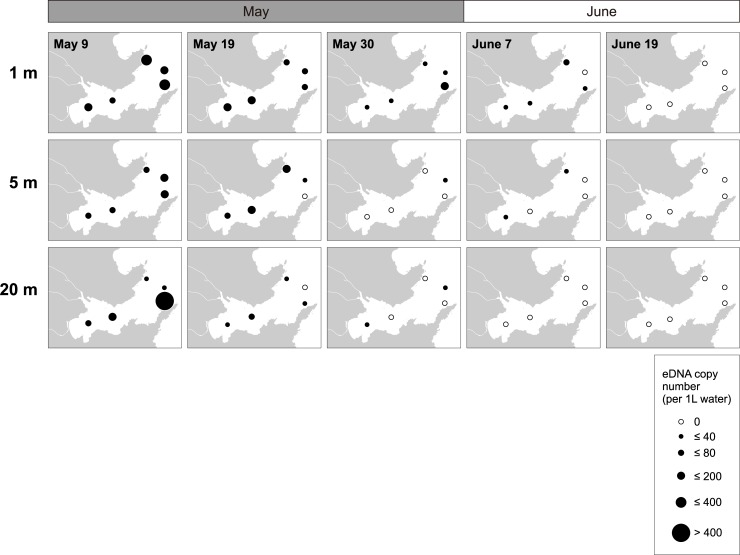
Changes in the vertical distribution of juvenile chum salmon eDNA at one-, five- and twenty-meter depths in Otsuchi Bay from early May to mid-June in 2017. White circles present no chum salmon eDNA was detected. The sizes of black circles correspond to the copy numbers. Refer Fig 4 for the detailed locations and names of the sampling sites.

For the five sampling sites in the central bay and bay mouth, the vertical distribution of chum salmon eDNA was also investigated. Chum salmon eDNA was detected at almost all depths examined until the middle of May (copy number = 14.0–1041.1; Fig 6; S2 Data), but from late May, the chum salmon eDNA was frequently detected at shallow layers than at deep layers in many sites although any comparisons among depths were not statistically significant (two-way ANOVA, *P*>0.05; S3 Fig). In the middle of June, chum salmon eDNA was undetectable at all depths (Fig 6).

**Fig 7:**
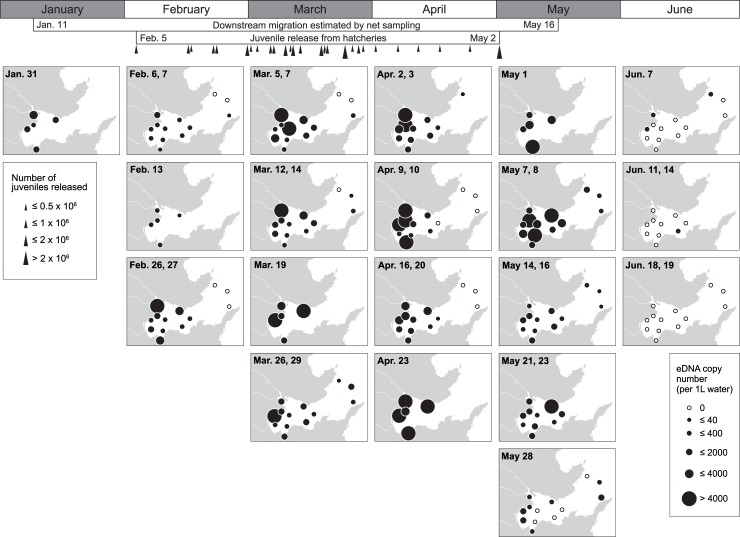
Changes in the horizontal distribution of juvenile chum salmon eDNA in Otsuchi Bay from late January to mid-June in 2018. Months are indicated at the top of the panels. The periods of downstream migration in rivers and juvenile release from hatcheries were presented below the months. Triangles below the period showing the juvenile release present the timing and number of juveniles released from two hatcheries. Sampling dates are shown at the top left of each panel and time goes from top to down in each month. In the case that two sampling dates are indicated, water collection was performed in the same week and the results of the samples collected from the shorefront and bay sites were demonstrated in a single panel. White circles present no chum salmon eDNA was detected. The sizes of black circles correspond to the copy numbers. Refer Fig 4 for the detailed locations and names of the sampling sites.

**Fig 8:**
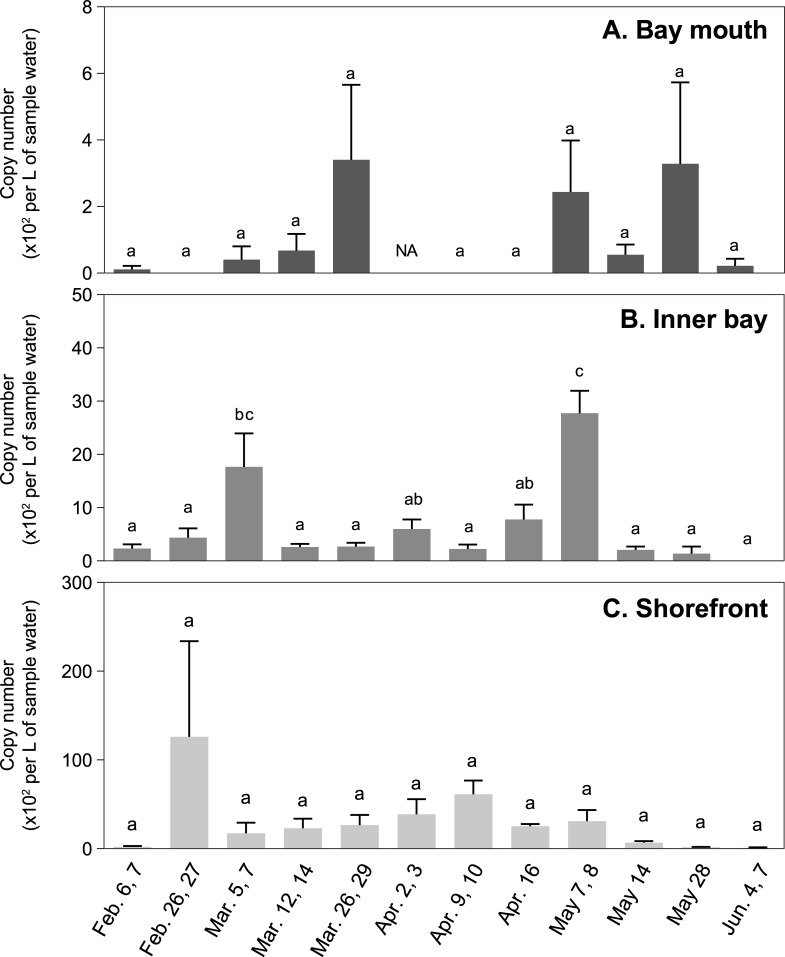
Average density of chum salmon eDNA in the bay mouth, inner bay and shorefront from early February to early June in 2018. Stn. e1, Ex.1 and e2 were grouped as ‘bay mouth’ (A), Stn. e3, e4, 9, 11 and 2 as ‘inner bay’ (B), Hakozaki, Murohama, Murohama fishing port, the river mouth of the Otsuchi River and Hyotan Island as ‘shorefront’ (C). Each bar indicates the average of copy numbers detected in each category. One-way ANOVA followed by the Tukey’s multiple comparisons was performed and statistical significance (*P*<0.05) among different time points were indicated by different alphabets.

In 2018, more comprehensive research was conducted from late January to the middle of June for five months. Downstream migration of juvenile chum salmon was already found in the middle of January in the Koduchi and Unosumai Rivers (Kawakami et al. in prep.), although juvenile release from hatcheries was not started yet (S2 Table). In accord with this fact, chum salmon eDNA was detected in Otsuchi Bay from late January (Fig 7; S3 Data). The chum salmon eDNA was considerably increased from late February at the shorefront sites. While fluctuating in the amount of eDNA, the high levels of chum salmon eDNA at the shorefront sites continued until early May (Fig 7). This increase in chum salmon eDNA matched with the juvenile release from hatcheries, which was started from February 5 and continued until May 2 (Fig 7 and S2 Table). In March, chum salmon eDNA was also detected at bay mouth sites (Stn. e1, e2 and Ex.1). In April, however, no detectable amount of chum salmon eDNA was found at the bay mouth sites, even though high copy numbers of chum salmon eDNA were found at the shorefront sites. In early May, chum salmon eDNA was again detected at the bay mouth sites. From the middle of May, the chum salmon eDNA became less detectable except for the Hyotan Is. site in late May (May 21 and 23), and even less in the end of May and early June. In late June, no chum salmon eDNA was detected in Otsuchi Bay (Fig 7). When the sampling sites were grouped into three areas, bay mouth (N = 3), inner bay (N = 5) and shorefront (N = 5), the Chi-square analysis revealed that the observed changes in chum salmon eDNA levels did not randomly occur (*P*<0.0001). Therefore, we hypothesized that the chum salmon eDNA distribution changes in particular patterns, and carried out one-way ANOVA followed by the Tukey's comparisons to analyze the temporal changes of the average chum salmon eDNA density in those three areas. The statistical analysis detected two significant peaks in early March and early May in the inner bay (Fig 8). There were also three peaks in the bay mouth in late March, early May and late May, but they were not significant (Fig 8). Sea surface temperature and salinity during the survey period was between 6.1–15.7°C and 26.9–33.4‰, respectively. Those at 1-m depth were 5.9–14.2°C and 29.3–33.8‰, respectively. At both depths, the temperature was the lowest in March and increased until June.

## Discussion

In the present study, we developed an eDNA method and examined the spatiotemporal distribution of juvenile chum salmon eDNA in Otsuchi Bay, a small closed bay typical on the Sanriku-ria Coast. Our aquarium experiments using juvenile chum salmon demonstrated a strongly-positive correlation between the number of juveniles and eDNA copy number, implying that the observed changes in chum salmon eDNA represent the spatiotemporal distribution of juvenile chum salmon. The downstream-migrated chum salmon juveniles grow in the Sanriku bays for a few months before outmigration to open ocean. Previous studies showed that growth and survival rate of juveniles in the coastal period are critical for the adult returning rate of chum salmon [10,29]. The large-scale spatiotemporal survey of salmon eDNA, therefore, provides an important basis to understand the ecophysiology of juvenile chum salmon in the Sanriku bays.

### Methodological consideration

In general, eDNA analyses can be categorized into two methods: a DNA metabarcoding by next generation sequencing for whole communities/fauna existing, and a quantitative method using a real-time PCR that targets a particular species. In the current study, we aimed to perform large-scale sampling (over 600 samples in the 2018 spring) to investigate the dynamics of the spatiotemporal distribution of chum salmon juvenile and thus selected the quantitative real-time PCR method. Our established method is specific and quantitative that does not detect DNA of other salmonid species such as masu salmon that co-occur with chum salmon in the northern Japan’s mainland. The amount of chum salmon eDNA showed a positive Pearson’s correlation to the number of juveniles (Fig 3), which is similar to the linear model by Yamamoto et al. (2016). When the amount of eDNA in an aquarium was converted to the eDNA amount released from a fish per unit time, the released eDNA from a single fish was nearly equal when density was low to middle (3 to 30 fish per 50 L water). On the other hand, more eDNA was released from fish in higher density conditions (60 and 120 fish per 50 L water) (Fig 3). This result indicates that the amount of eDNA released from animals is dependent on their physiological and ecological conditions. In a high-density environment, inter-individual contact such as aggressive behaviors of juvenile chum salmon [30] may increase, which leads to increase in deciduous scales and mucus secretion. In nature, such a high density rarely occurs but careful attention must be required, because possible scenarios like school formation of juveniles could result in a high fish density.

Although our quantitative protocol successfully detected salmon eDNA in the laboratory conditions, it could not detect from the field samples initially, probably due to the inhibitory substance(s) in water of Otsuchi Bay. PCR inhibitions in which PCR amplification fails or delays have been reported in environmental samples from lentic and lotic systems [31]. More than half of our samples were collected from the surf-zone, therefore the humic and phenolic compounds, which are two known PCR inhibitors in environmental water samples [32], were likely to be contained in the samples. Moreover, the Sanriku Coast including Otsuchi Bay were severely damaged by the 2011 off Pacific coast of Tohoku Earthquake and subsequent tsunami, which flooded a large amount of land-originated substances into the Sanriku bays. In addition, intensive post-disaster construction and damage repair are in progress. The repeated disturbances of sediments may have increased the PCR inhibitor contents in the water. Our results showed that the combination of the additional cleanup and the modified PCR master mix overcame the inhibition problems. PCR inhibition has not been reported in previous field studies [33–35], however wide variations can exist PCR inhibition in different aquatic systems and environments.

The eDNA amount in water is a balance between the release and decomposition of DNA. Our time-course experiment showed that the amount of eDNA was significantly decreased even before fish removal at all temperatures examined (Fig 2), implying rapid degradation of the released eDNA in water. The decrease in salmon eDNA was slower at lower temperatures, which may be due to a slower enzymic degradation, leading to a longer half-life of DNA at lower temperatures [36–38]. On the other hand, DNA could be released less at higher temperatures, since chum salmon is a typical cold-water fish and a high temperature such as 20°C is not suitable for their metabolism and mobility as suggested by previous studies using other fish species [18,19]. These results showed that water temperature determines the eDNA amounts in various ways, and we should consider and integrate other physical parameters when applying the eDNA technique for the estimation of the salmon abundance in the field.

Another cause for DNA decomposition in water is thought to be microbes. Since the pore size of glass fiber filter used in this study (0.7 μm) was larger than those regularly used for microbes (0.22 μm), the observed changes in the bacterial abundance were possibly a representation of particle-attached microbes. Even though our data did not show the entire microbial community, the increase in the bacterial DNA corresponded well with the decrease of salmon eDNA (Figs 1 and 2), suggesting that bacteria play a key role in the eDNA degradation. A similar interaction was reported by Lance et al. [38] in which a microbial boost remarkably reduced total eDNA, suggesting that bacteria could, probably enzymatically, contribute to eDNA decay [37,39]. In our study, we used artificial seawater in the aquarium experiments. Therefore, the initial microbial amounts, dynamics and composition are highly different from those of natural seawater. However, salmon eDNA became undetectable within three days after fish removal even at a low temperature (8°C; Fig 2), suggesting that eDNA clearance in natural seawater is fast.

Most aquarium eDNA experiments in previous studies were performed using unfed animals to avoid the fecal discharge since it is generally considered as one of the main sources of eDNA in nature [40,41]. This allowed to expect that the eDNA amount should be larger in the water of the fed individuals than that of the fasted ones. However, except for the day 1, there was no notable differences in the juvenile chum salmon eDNA amounts between the two groups (Fig 1). Although the reason for higher salmon eDNA in the fasted group on day 1 than that of the fed group is unknown, our results indicate that most chum salmon eDNA was not derived from feces. Body fluids and skin mucus are possible major origins of eDNA as suggested by Barnes & Turner [41]. Moreover, juvenile chum salmon displays aggressive behaviors such as snipping and chasing other individuals, and it was often observed that scales were taken off when the fish was snipped [30] and floated in the experimental tanks, which consequently increased the eDNA contents in the holding water. Future study is needed to elucidate the origins of juvenile chum salmon eDNA.

### Spatiotemporal distribution of chum salmon eDNA in Otsuchi Bay

We showed the spatiotemporal distribution of juvenile chum salmon in Otsuchi Bay by the eDNA analyses. The salmon eDNA appeared in shallow waters in the bay from late January to the middle of June, and became abundant from March to early May, which corresponded to the timing of juvenile release from hatcheries and their downstream migration estimated by net capture in rivers (Figs 5 and 7). It is noteworthy that chum salmon eDNA was already detected from January in 2018, which is earlier than the timing of juvenile release from hatcheries. Although a part of chum salmon eDNA detected may have originated from the spawning adults that were still in the rivers, downstream migration of juvenile chum salmon was already found in the middle of January in the Koduchi and Unosumai Rivers in 2018 (Kawakami et al. in prep.), indicating that salmon eDNA found in the bay in January came from the juveniles derived from natural spawning as observed in the Otsuchi River [42].

The current large-scale survey showed that the main nursery habitat of juvenile chum salmon after sea entry appeared to be the shorefront and beach areas of the inner part of the bay like the river mouth of the Otsuchi Rivers and Murohama, where waves and high salinity of waters of open ocean are less influential. In fact, body size of juvenile chum salmon collected by surf-zone and beach seine net towing in Otsuchi Bay in the spring of 2017 was relatively small, ranging from 35 to 45 mm FL (Kawakami et al. unpublished data), suggesting that small juveniles inhabit the beach area soon after sea entry. In March, juveniles seemed to spread over the bay and to move offshore. In the middle of May, the amount of chum salmon eDNA dramatically decreased in the shorefront area and the eDNA disappeared in the bay by late June (Figs 5 and 7), indicating that the outmigration to open ocean is completed by late June. This seasonal habitat shifts observed in Otsuchi Bay were also reported in other bays in Sanriku such as Kesennuma, Koizumi and Shizugawa Bays and Hokkaido by juvenile collection [43–45].

In addition, our eDNA data indicated the existence of two timings in offshore migration of juvenile chum salmon, March–early April and May–June (Figs 7 and 8). Two significant peaks in chum salmon eDNA in the inner bay were detected in early March and early May. The salmon eDNA levels also tended to increase in the bay mouth in late March and late May, which were lagged behind the increase in the inner bay. Kaeriyama [44] reported two outmigrating juvenile groups in the Sanriku area, in which the early group migrated offshore when the Oyashio Current approached the coast, while the late group migrated after its retreat. The Oyashio Current transports cold, less-saline and nutrient-rich subarctic waters from the north, and strongly influences the oceanographic condition on the Sanriku coast, especially in spring. Satellite and our own CTD observations in 2018 captured the cold water of lower than 7°C coming to the Sanriku Coast in March and its replacement with warmer water in May, indicating the Oyashio Current dynamics. This supports our idea that the present eDNA data could detect the outmigration of the early and late groups. The intrusion of the cold and nutrient-rich water brought the Oyashio zooplankton community to Otsuchi Bay and also triggered phytoplankton blooms [46]. Furthermore, the changes in food preference with growth were suggested by the zooplankton species composition in the stomach contents of juvenile chum salmon [44]. Future multiplex analysis on eDNA of zooplanktons and chum salmon will clarify the relationship between juvenile chum salmon behavior and zooplankton dynamics.

The present study developed an eDNA method to investigate the chum salmon ecology and demonstrated the spatiotemporal distribution of juvenile chum salmon in Otsuchi Bay. Some basic characteristics of chum salmon eDNA were studied in controlled laboratory conditions for the extrapolation to field analyses and we successfully optimized the qPCR-based eDNA assay protocol for natural seawater samples. Further research is, however, needed to estimate the biomass and population dynamics accurately in nature, because natural environment is more complex than the laboratory, such as tide, diurnal movement, status and origins of eDNA in nature, and other factors that influence eDNA like UV and pH. In addition, continuous but irregular juvenile release from hatcheries (S1 and S2 Tables) makes it difficult to estimate the spatiotemporal movement of juvenile in the bay. In spite of these, the current study succeeded to detect the two plausible outmigrating groups of chum salmon juvenile in March and May by using eDNA, strongly implying that the eDNA technique is a powerful to allow large-scale and comprehensive research to understand the spatiotemporal dynamics of juvenile chum salmon without affecting populations, in cooperation with traditional fieldwork.

## Supporting information

S1 Data Spread sheet containing the raw data (copy numbers per L of sample water) from the 2017 spring (horizontal). (CSV)

S2 Data Spread sheet containing the raw data (copy numbers per L of sample water) from the 2017 spring (vertical). The column 'Group' is the categories for statistical analyses.
(CSV)

S3 Data Spread sheet containing the raw data (copy numbers per L of sample water) from the 2018 spring. The column 'Group' is the categories for statistical analyses.
(CSV)

S1 Fig Sequence variations of *Oncorhynchus keta*, *O*. *masou*, *O*, *mykiss* and *Salmo trutta* in the target region of the chum salmon qPCR. The hybridizing sites of the forward and reverse primers and probe for chum salmon DNA are shown at the top of the line. Shaded sites indicate the mismatches among the three salmonid species that co-occur with chum salmon in the northern Japan’s mainland.
(EPS)

S2 Fig PCR inhibition in the eDNA samples from Otsuchi Bay. A, PCR amplification curves of the serially diluted eDNA samples spiked with chum salmon DNA (csDNA) showing the PCR inhibitory strength of the water of Otsuchi Bay. The eDNA was extracted with DNeasy Blood and Tissue kit (Qiagen) and were serially diluted. ABI TaqMan Universal Master Mix II was used for the PCR amplification. B, Real-time PCR amplification curves showing the combined effects of two types of PCR master mixes and additional purification on freeing the PCR inhibitors from the field samples of Otsuchi Bay. Two types of PCR reagents (ABI TaqMan Universal Master Mix II + BSA [final conc. 1 mg/mL] and ABI TaqMan Environmental Master Mix 2.0) were tested and Environmental Master Mix displays a higher tolerance against PCR inhibition. Additional purification by DNeasy PowerClean Pro Cleanup kit (Qiagen) improved the sample quality to give identical amplification efficiency as in control csDNA.
(EPS)

S3 Fig Average density of chum salmon eDNA at different layers from May to June in 2017. Each bar indicates the average copy numbers detected at each depth. One-way ANOVA followed by the Tukey’s multiple comparisons was performed and statistical significance (*P*<0.05) among different time points were indicated by different alphabets.
(EPS)

S1 Table Dates and number of juveniles released from Otsuchi and Unosumai hatcheries in the spring of 2017. (XLSX)

S2 Table Dates and number of juveniles released from Otsuchi and Unosumai hatcheries in the spring of 2018. (XLSX)
